# Signal regulators of systemic acquired resistance

**DOI:** 10.3389/fpls.2015.00228

**Published:** 2015-04-13

**Authors:** Qing-Ming Gao, Shifeng Zhu, Pradeep Kachroo, Aardra Kachroo

**Affiliations:** ^1^Department of Plant Pathology, University of KentuckyLexington, KY, USA; ^2^Department of Plant Biology and Ecology, College of Life Sciences, Nankai UniversityTianjin, China

**Keywords:** systemic resistance, plant defense, glycerol-3-phosphate, lipids, reactive oxygen species

## Abstract

Salicylic acid (SA) is an important phytohormone that plays a vital role in a number of physiological responses, including plant defense. The last two decades have witnessed a number of breakthroughs related to biosynthesis, transport, perception and signaling mediated by SA. These findings demonstrate that SA plays a crictical role in both local and systemic defense responses. Systemic acquired resistance (SAR) is one such SA-dependent response. SAR is a long distance signaling mechanism that provides broad spectrum and long-lasting resistance to secondary infections throughout the plant. This unique feature makes SAR a highly desirable trait in crop production. This review summarizes the recent advances in the role of SA in SAR and discusses its relationship to other SAR inducers.

## Introduction

Plants being sessile are constantly exposed to a number of pathogenic microbes, which based on their infectious lifestyles can be broadly divided into biotrophs and necrotrophs (Glazebrook, 2005; Mengiste, 2012; Lai and Mengiste, 2013). Biotrophic pathogens rely on nutrients from living host cells, whereas necrotrophic pathogens feed on dead cells. Plants employ distinct immune responses to counter these pathogens and this aspect has been covered in detail in several recent reviews (Spoel and Dong, 2012; Dangl et al., 2013). This first layer of host defense involves the recognition of pathogen (or microbe) associated-molecular patterns (PAMPs/MAMPs), such as bacterial flagellin, lipopolysaccharides, and peptidoglycans. PAMPs are recognized by specialized transmembrane proteins in the plant, termed pattern recognition receptors (PPRs). PRR-mediated recognition of PAMPs triggers downstream signaling leading to the activation of basal resistance termed PAMP-triggered immunity (PTI; Schwessinger and Ronald, 2012). PTI can be suppressed by pathogen encoded effector proteins commonly known as avirulence (*avr*) factors (Göhre and Robatzek, 2008; Cunnac et al., 2009; Bozkurt et al., 2011; Caillaud et al., 2012; Marrtin and Kamoun, 2012; Cheong et al., 2013; Cui et al., 2013; Dangl et al., 2013; Giraldo and Valent, 2013). The avr factors are in turn recognized by the host encoded resistance (R) proteins, which confer more durable and robust resistance termed *R* gene- or effector-triggered immunity (ETI; Bogdanove and Martin, 2000; Mackey et al., 2002, 2003; Gu et al., 2005; Jones and Dangl, 2006; Narusaka et al., 2009; Cesari et al., 2013). ETI is generally associated with programmed cell death (PCD) at the site of infection and this phenomenon is called hypersensitive response (HR; Holliday et al., 1981; Dangl et al., 1996; Morel and Dangl, 1997).

Induction of local responses is associated with the transport of defense signals throughout the plant resulting in broad-spectrum disease resistance against secondary infections. This phenomenon, known as systemic acquired resistance (SAR), is conserved among diverse plants and confers long-lasting resistance to unrelated pathogens (Chaturvedi et al., 2008; Dempsey and Klessig, 2012; Fu and Dong, 2013; Kachroo and Robin, 2013; Lucas et al., 2013; Shah and Zeier, 2013; Wendehenne et al., 2014). Several studies have shown that the establishment of SAR involves the generation and transport of signals via phloem to the uninfected distal tissues (Guedes et al., 1980; Tuzun and Kuc, 1985). Among the signals contributing to SAR are salicylic acid (SA) and several components of the SA pathway including the methylated derivative of SA (methyl SA,MeSA, Park et al., 2007). Additionally, the diterpenoid dehydroabietinal (DA, Chaturvedi et al., 2012), the nine carbon (C9) dicarboxylic acid azelaic acid (AzA, Jung et al., 2009), an amino acid derivative pipecolic acid (Pip; Návarová et al., 2012), auxin (Truman et al., 2010), the phosphorylated sugar glycerol-3-phosphate (G3P, Chanda et al., 2011; Mandal et al., 2012; Yu et al., 2013), the free radicals nitric oxide (NO) and reactive oxygen species (ROS; Wang et al., 2014a; El-Shetehy et al., 2015), galactolipids (Gao et al., 2014), factors contributing to cuticle formation (Xia et al., 2009, 2010, 2012) and the lipid transfer proteins (LTPs) DIR1 (Defective in Induced Resistance, Maldonado et al., 2002) and AZI1 (AzA insensitive, Jung et al., 2009), have all been proposed to serve as SAR signals. Here, we review the role of SA in SAR and discuss its relationship to these various SAR signals.

## SA Biosysnthesis and SAR

**FIGURE 1:**
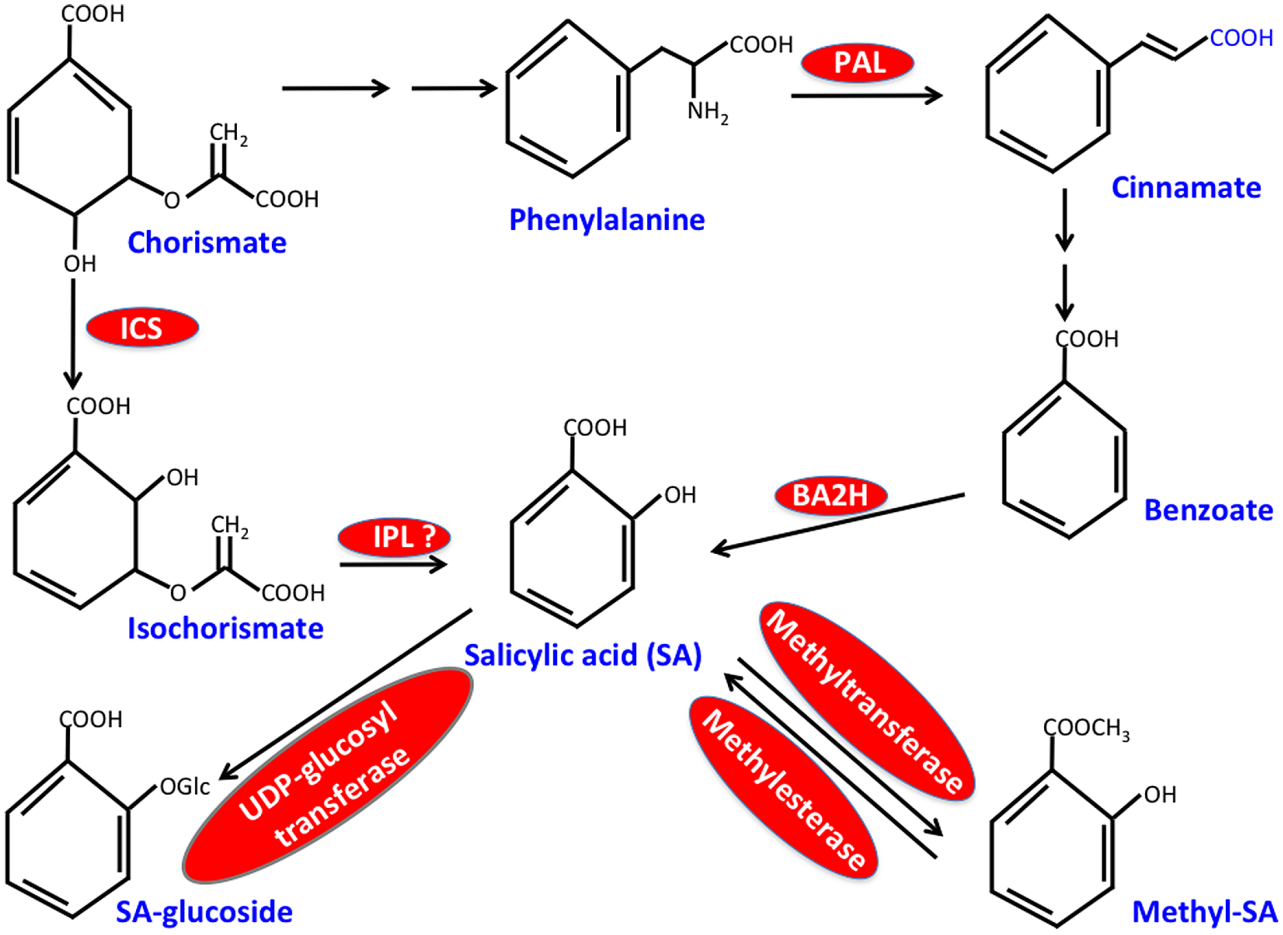
**Simplified scheme for salicylic acid (SA) biosynthesis in plants**. Critical enzymes are shown in red. ICS, isochorismate synthase; IPL, isochorismate pyruvate lyase; PAL, phenylalanine ammonia-lyase; BA2H, benzoic acid 2-hydroxylase.

Salicylic acid biosynthesis occurs via the shikimic acid pathway, which forms two distinct sub-branches both of which synthesize SA. These branched pathways, designated as isochorismate synthase (ICS)- and the phenylalanine ammonia-lyase (PAL)-derived pathways, utilize chorismate as the common precursor (Shah, 2003; Chen et al., 2009; Kachroo and Kachroo, 2009; Yu et al., 2010; An and Mou, 2011; Dempsey et al., 2011; Vlot et al., 2009; Singh et al., 2013; **Figure 1**). The first step of the PAL pathway involves conversion of phenylalanine (Phe) to *trans*-cinnamic acid and this reaction is catalyzed by PAL, a key enzyme of this pathway that is induced by pathogen infection. The *Arabidopsis* genome encodes four *PAL* isoforms and *PAL* quadruple mutants or wild-type plants treated with the PAL inhibitor, 2-aminoindan-2-phosphonic acid contain reduced SA, show increased susceptibility to pathogens and are unable to induce SAR (Yalpani et al., 1993; Mauch-Mani and Slusarenko, 1996; Pallas et al., 1996; Huang et al., 2010). Although relative contributions of PAL versus ICS branches toward SA biosynthesis vary between different plant species, at least in *Arabidopsis* majority of the pathogen-induced SA appears to be derived from the ICS branch. The ICS branch involves conversion of chorismate to isochorismate by ICS followed by coversion of isochorismate to SA by isochorismate pyruvate lyase (IPL). The *Arabidopsis* genome encodes two isoforms of ICS, of which ICS1 (SID2) accounts for ∼95% of basal- or pathogen-induced SA (Strawn et al., 2007; Garcion et al., 2008). A mutation in *ICS1* also impairs SAR (Wildermuth et al., 2001; Jung et al., 2009; Chanda et al., 2011; Wang et al., 2014a), suggesting that SA contributed by both PAL- and ICS-pathways is critical for the induction and/or establishment of SAR. This together with the compromised SAR phenotype of transgenic plants expressing bacterial salicylate hydroxylase (NahG; Vernooij et al., 1994), an enzyme that catalyzes the conversion of SA to catechol, reemphasize the importance of SA in SAR. It is unclear what factors govern the specific recruitment of the PAL or ICS pathways for SA biosynthesis.

Salicylic acid synthesized in the chloroplasts is exported out to the cytosol via EDS5, a member of the MATE (Multidrug and Toxin Extrusion) transporter family, located in the chloroplast envelope (Nawrath et al., 2002; Serrano et al., 2013). Notably, a mutation in *EDS5* results in complete shut down of SA biosynthesis rather than SA accumulation within the chloroplasts. Thus, mutations in *ICS1* and *EDS5* similarly affect SA levels and the corresponding mutants thereby exhibit overlapping defense defects. This is likely due to negative feed-back regulation of ICS1 by SA (Fragnière et al., 2011; Serrano et al., 2013). The triphosphate tunnel metalloenzyme 2 is a negative regulator of the SA feed-back loop and functions in defense signal amplification (Ung et al., 2014). Pathogen induced expression of *ICS1* requires the binding of calmodulin binding protein CBP60g and its homolog, non-calmodulin binding SARD1 (SAR Deficient 1) to the *ICS1* promoter. CBP60g and SARD1 specifically bind the GAAATTTTGG sequence in the ICS1 promoter (Truman and Glazebrook, 2012). The induction of *ICS1* and thereby SA biosynthesis is inhibited in *cbp60g sard1* double mutant, resulting in compromised SAR (Zhang et al., 2010).

Although a number of studies have demonstrated the critical requirement of SA in SAR, a specific requirement for SA accumulation beyond basal levels during SAR has not been established. For instance, plants lacking a functional R protein RPS2 accumulate normal levels of SA in their distal tissues in response to infection by *Pseudomonas syringae pv. tomato* expressing *avrRpt2,* yet these plants are compromised for SAR (Cameron et al., 1999). Additionally, exogenous application of either G3P or AzA, which induce SAR in wild-type plants, do not induce SA accumulation. However, neither G3P nor AzA can confer SAR in *ics1* (*sid2*) mutant plants, which contain significantly reduced basal- and pathogen-induced SA. Thus, although SA is clearly critical for SAR, accumulation of SA alone is insufficient to establish SAR. Furthermore, although SA has been shown to accumulate to varying levels in the distal tissues of SAR induced plants (**Table 1**), there is no evidence suggesting that this accumulation is essential for SAR.

**Table 1:** Free and bound salicylic acid (SA) levels reported in distal tissues of mock-and pathogen-inoculated plants.

Free SA (ng/gFW)		Bound SA (SAG; ng/gFW)		Pathogen used, and concentration	Host	HPl^a^	Analytical procedure used	Reference
Mock	SAR- induced tissues	Mock	SAR- induced tissues					
∼80	∼1250	NA^b^	NA	*Psm^d^ ES4326* expressing *AvrRpm1,* OD_600_ = 0.01	*Arabidopsis*	48	GC-MS^g^	Attaran et al. (2009)
∼200	∼2600	NA	NA	*Pst^e^ DC3000 expressing AvrRpt2,* 1 × 10^7^ CFU^f^/ml	*Arabidopsis*	48	HPLC^h^	Singh et al. (2013)
∼ 50–150	∼370–1200	∼100–250	∼300–1000	Tobacco mosaic virus	Tobacco	144	HPLC	Shulaev et al. (1995)
∼30–70	∼30–300	NA	NA	*Psm DC3000 expressing AvrRpml,* 5 × 10^7^ CFU/ml	*Arabidopsis*	48	HPLC	Kiefer and Slusarenko (2003)
∼100	∼450	NA	NA	*Psm ES4326* expressing *AvrRpml,* OD_600_ = 0.02	*Arabidopsis*	48	GC-MS	Mishina and Zeier (2006)
∼5.1	∼21.3	ND^c^	∼287.3	*Pseudomonas lachrymans,* 4 × 10^8^ CFU/ml	Cucumber	120	HPLC	Meuwly and Métraux (1993)
∼70	∼200	∼130	∼400	*Pst DC3000 expressing AvrRpt2,* 10^7^ CFU/ml	*Arabidopsis*	48	HPLC	Lawton et al. (1995)
∼160	∼400	∼1300	∼1800	*Pst DC3000 expressing AvrRpt2,*	*Arabidopsis*	48	HPLC	Gao et al. (2014)
∼420	∼500	∼500	∼1600	*Coronatine-deficient Psm expressing AvrRpt2*, 1 × 10^6^ CFU/ml	*Arabidopsis*	48	HPLC	Liu et al. (2011)
∼80	∼100	∼470	∼700	*Pst DC3000 expressing AvrRpt2,*	*Arabidopsis*	48	HPLC	Cameron et al. (1999)
∼35	∼60	∼700	∼1600	*Pst DC3000 expressing AvrRpt2,* 1 × 10^6^ CFU/ml	*Arabidopsis*	48	HPLC	Chanda et al. (2011)
∼220	∼300	∼210	∼1200	*Coronatine-deficient Psm expressing*	*Arabidopsis*	60	HPLC	Liu et al. (2010)
∼40	∼60	∼450	∼800	*Pst DC3000 expressing AvrRpt2,* 1 × 10^6^ CFU/ml	*Arabidopsis*	48	HPLC	Wang et al. (2014a)
∼40	∼65	∼600	∼1600	*Pst DC3000 expressing AvrRpt2,* 1 × 10^6^ CFU/ml	*Arabidopsis*	48	HPLC	Xia et al. (2009)
∼32–52	∼51–83	∼70–164	∼82–196	Tobacco mosaic virus	Tobacco	168	HPLC	Vernooij et al. (1994)
∼40	∼90	∼20	∼85	Tobacco necrosis virus	Cucumber	53	HPLC	Molders et al. (1994)

*^a^HPI, hour post infection; ^b^NA, not available; ^c^ND, not detected; ^d^Psm, P. syringae pv *maculicola; ^e^Pst, P. syringae* pv *tomota;*^f^CFU, colony-forming unit; ^g^GC-MS, gas chromatography-mass spectrometry; ^h^HPLC, high performance liquid hromatography*.

In comparison to local tissues, the distal tissues of SAR-induced plants have been shown to accumulate a broad range of SA ranging from as low as 10 ng/ g FW to ∼2.6 μg/g FW (**Table 1**; Rasmussen et al., 1991; Yalpani et al., 1991; Meuwly and Métraux, 1993; Molders et al., 1994; Vernooij et al., 1994; Lawton et al., 1995; Shulaev et al., 1995; Cameron et al., 1999; Kiefer and Slusarenko, 2003; Mishina and Zeier, 2006; Attaran et al., 2009; Liu et al., 2010, 2011; Xia et al., 2010; Chanda et al., 2011; Gao et al., 2014). The inability to accumulate SA in distal tissues has also been suggested to contribute to impaired SAR in *ald1* (*agd2*-Like Defense response protein 1) and *fmo1* (Flavin Monooxygenase 1) mutants, both of which accumulate normal SA in the local tissue (Song et al., 2004a,b; Mishina and Zeier, 2006). ALD1 encodes an aminotransferase that catalyzes the biosynthesis of the SAR inducer Pip, (Song et al., 2004b; Návarová et al., 2012) and FMO1 has been suggested to function downstream of Pip (Návarová et al., 2012). Thus, other factors besides SA might contribute to the SAR defect of *ald1* and *fmo1* mutants. One possibility is that SAR can be induced via SA-independent factors so long as a minimum basal level of SA can be maintained. Alternatively, SA accumulation in distal tissues might contribute to the priming process resulting in the activation of stronger defense responses upon secondary infections (Návarová et al., 2012; Gruner et al., 2013).

## SA-Derivatives and SAR

A majority of the synthesized SA is converted and stored as biologically inactive derivatives via glucosylation, methylation and amino acid conjugation since accumulation of the acidic SA has adverse physiological consequences (Heil and Baldwin, 2002; Heidel et al., 2004). These include SA 2-*O*-β-D-glucose (SAG), SA glucose ester (SGE), methyl SA (MeSA), and SA-amino acid conjugates (Pierpoint, 1994; Vlot et al., 2009; Dempsey et al., 2011). Most recently, SA was shown to be derivatized to 2,3-dihydroxybenzoic acid (2,3-DHBA) and this reaction is catalyzed by SA 3-hydroxylase (S3H; Zhang et al., 2013). As predicted *s3h* knockout plants contain very high levels of SA, while plants expressing *S3H* gain-of-function mutations accumulate high amounts of 2,3-DHBA (Zhang et al., 2013). SA derivatives serve as storage forms that can be converted back to free SA (Hennig et al., 1993; Kawano et al., 2004; Kachroo and Kachroo, 2012). With the exception of MeSA however, the exact role of SA derivatives in SAR remains unclear.

Methyl SA is a volatile and phloem mobile SA derivative, which accumulates in infected and distal tissues in response to pathogen infection. Like MeSA, SA also accumulates in the phloem of tobacco leaves infected with tobacco mosaic virus or *Colletotrichum lagenarium* and in cucumber leaves infected with tobacco necrosis virus (Malamy et al., 1990; Métraux et al., 1990; Park et al., 2007). For SAR, MeSA must be converted to SA in the distal tissues between the 48–72 h period post primary infection. This time-frame correlates with that of pathogen-inducible MeSA accumulation in infected and systemic tissues. The biosynthesis of MeSA is catalyzed by SA methyltransferases (SAMT/BSMT), and the conversion of MeSA back to SA is mediated by methyl esterase (MES; Chen et al., 2003; Effmert et al., 2005; Koo et al., 2007). The tobacco MES was first identified based on its ability to bind SA, and therefore designated as SA-binding protein 2 (SABP2; Kumar and Klessig, 2003). Grafting studies in tobacco plants silenced for SABP2 have shown that SABP2 activity in scions, but not root-stocks is required for normal SAR (Park et al., 2007). Furthermore, the synthetic SA analog, 2,2,2,2^′^-tetra-fluoroacetophenone, which inhibits the esterase activity of SABP2, also inhibits SAR (Park et al., 2009). As in tobacco, homologs of SABP2 (AtMES9) and SAMT AtBSMT1 are required for SAR in *Arabidopsis* (Liu et al., 2011). Thus, the ability to derivatize SA to MeSA and reconvert MeSA back to SA are critical for SAR. Intriguingly, the requirement for *AtBSMT1* in SAR can be bypassed by prolonged exposure to light after pathogen inoculation (Attaran et al., 2009; Liu et al., 2011). However, the role of light signaling in SAR and how it might compensate for MeSA is unclear. It is also not known whether MeSA merely functions to deliver SA to the distal tissues or has other function(s) in SAR. Notably, a certain percentage of SA is always transported from the inoculated to distal tissues (Meuwly et al., 1995; Kiefer and Slusarenko, 2003). The biological significance of this transport is unclear, particularly in view of the fact that SA is not considered to be the mobile SAR signal since wild-type tobacco scions grafted onto NahG root-stocks exhibit normal SAR (Vernooij et al., 1994; Meuwly et al., 1995; Kiefer and Slusarenko, 2003).

## Regulation of SA Accumulation and SAR

Besides proteins that directly contribute to SA biosynthesis (ICS and PAL) or transport (EDS5), a number of other proteins have been identified that participate in pathogen induced SA accumulation and thereby SAR. These include EDS1 (Enhanced Disease Susceptibility 1), PAD4 (Phytoalexin Deficient 4), and NDR1 (Non-race-specific Disease Resistance 1; Century et al., 1995, 1997; Falk et al., 1999; Jirage et al., 1999; McDowell et al., 2000; Feys et al., 2001; Coppinger et al., 2004; Ishihara et al., 2008; Bhattacharjee et al., 2011; Cacas et al., 2011; Heidrich et al., 2011; Knepper et al., 2011; Lu et al., 2013). Unlike *ICS1* and *EDS5*, mutations in *EDS1*, *PAD4*, or *NDR1* cause partial reduction in SA levels. EDS1 and PAD4 are lipase-like proteins, which together with another lipase-like protein SAG101 (Senescence Associate Gene 101) form binary and ternary complexes (Feys et al., 2005; Zhu et al., 2011). EDS1 interacts with PAD4 in both cytosol and nucleus, and with SAG101 only in the nucleus. EDS1, PAD4, and SAG101 function cooperatively as well as independently in pathogen defense (Feys et al., 2005; Venugopal et al., 2009; Rietz et al., 2011; Zhu et al., 2011). For instance, all three proteins are required for R-mediated resistance against Turnip crinkle virus (TCV) but only PAD4 is required for the SA-mediated induction of the *R* gene which confers HR against TCV (HRT; Chandra-Shekara et al., 2004, 2006, 2007). Interestingly, EDS1, but not PAD4 or SAG101, interacts with HRT and potentiates HRT-mediated HR to TCV (Zhu et al., 2011). Similarly, only PAD4 is required for resistance to the green peach aphid, whereas EDS1 and SAG101 are not (Pegadaraju et al., 2005, 2007; Louis et al., 2010, 2012). Both nuclear and extranuclear localization of EDS1 is important for its defense function (García et al., 2010). However, the role of binary or ternary complex formation between EDS1, PAD4, and SAG101 proteins remains unknown. EDS1 was recently shown to participate in both SAR signal generation in the local tissues as well as perception in the distal leaves (Breitenbach et al., 2014).

The *Arabidopsis* genome encodes two isoforms of EDS1 that function redundantly and can compensate for each other (Zhu et al., 2011). However, some *Arabidopsis* ecotypes, such as Wassilewskija, Landsberg, and Dujon, contain only one functional EDS1 isoform, and this is sufficient for normal resistance in these ecotypes. Like *Arabidopsis*, soybean also contains two EDS1 isoforms. Interestingly, *Arabidopsis eds1* mutant expressing the soybean *EDS1* orthologs is only partially restored in SA levels, but completely restored in bacterial resistance (Wang et al., 2014b). This further questions the requirement for increased SA accumulation during defense activation and raises the possibility that a certain threshold of SA may be sufficient to induce appropriate defense responses. The soybean *EDS1* orthologs are unable to potentiate TCV coat protein-derived activation of HRT even though they do interact with HRT (Wang et al., 2014b). This suggests that EDS1 orthologs in different plants may have evolved to perform overlapping as well as distinct functions.

## SA Signaling Components

Salicylic acid-mediated signaling leading to SAR is dependent on the ankyrin repeat containing protein NPR1 [Non-expressor of *Pathogenesis-Related* (*PR*) genes] (Dong, 2004). Under basal or uninduced conditions, NPR1 exists as a cytosolic inactive oligomer formed by intermolecular disulfide bonding (Mou et al., 2003). Reducing conditions resulting from accumulation of SA cause dissociation of the NPR1 oligomer into active monomers and the monomeric form of NPR1 is translocated into the nucleus (Kinkema et al., 2000; Mou et al., 2003; Tada et al., 2008). Nuclear localization of NPR1 facilitates its interaction with members of the TGACG motif binding (TGA) transcription factors that belong to the basic leucine zipper (bZIP) protein family (Zhang et al., 1999; Després et al., 2000; Niggeweg et al., 2000; Zhou et al., 2000; Chern et al., 2001; Fan and Dong, 2002; Kim and Delaney, 2002). This in turn enhances binding of the TGA factors to promoter elements of NPR1-dependent target genes (Wang et al., 2006, 2011). Like NPR1, TGA factors are also required for SAR; the *tga2 tga5 tga6* triple mutant is non-responsive to SA and is defective in SAR (Zhang et al., 2003). Recent studies have shown that NPR1 and TGA1 also undergo *S*-nitrosylation, which is necessary for the proper functioning of NPR1 in immunity and increases the DNA binding activity of TGA1 (Tada et al., 2008; Lindermayr et al., 2010). On the other hand, thiol *S*-nitrosylation has also been shown to promote NPR1 oligomerization and thereby its inactivation (Tada et al., 2008). The nuclear NPR1 is phosphorylated and degraded in a proteasome-dependent manner (Spoel et al., 2009), and the turnover of NPR1 is essential for SAR establishment. The *Arabidopsis* genome contains five paralogs of NPR1 (Liu et al., 2005). Like NPR1, NPR3, and NPR4 also interact with TGA proteins (Zhang et al., 2006). The *npr3 npr4* mutant plants accumulate elevated levels of NPR1 and are consequently defective in SAR. NPR3 and NPR4 bind SA and function as adaptors of the Cullin 3 ubiquitin E3 ligase to mediate NPR1 degradation in an SA-dependent manner (Fu et al., 2012). However, the two differ in that NPR3 has higher affinity for SA than NPR4, and SA promotes the NPR1–NPR3 interaction but inhibits the NPR1–NPR4 interaction. These contrasting effects might offer a possible explanation for the nuances underlying NPR1-dependent immunity under different levels of SA. For instance, high concentration of SA in infected tissues might favor binding of NPR3 with SA, which would mediate degradation of the cell-death suppressor NPR1, and initiate PCD and local immunity. On the other hand, lower SA levels in the distal uninfected tissue would minimize NPR3-SA binding, thereby inhibiting PCD. Interestingly, in yet another study, NPR1 was also shown to bind SA via the transition metal copper (Wu et al., 2012; Manohar et al., 2015). The binding of SA to NPR was suggested to induce a conformational change in NPR1 (Wu et al., 2012), which in turn is important for NPR1-dependant *PR1* expression.

NPR1 is also required for transgenerational SAR, which in turn involves epigenetic changes (Jaskiewicz et al., 2011; Luna et al., 2012). NPR1 othologs have been characterized from a number of plants including rice, tobacco, soybean, and cacao (Chern et al., 2001, 2005, 2014; Ekengren et al., 2003; Zwicker et al., 2007; Sandhu et al., 2009; Shi et al., 2010; Chen et al., 2013). Transgenic expression of *Arabidopsis NPR1* confers enhanced resistance in heterologous plants (Lin et al., 2004; Shi et al., 2010; Chen et al., 2012). Conversely, transgenic expression of soybean orthologs can complement the *Arabidopsis npr1* mutation (Sandhu et al., 2009). Overexpression of NPR1 also enhances pathogen resistance in monocots (Chern et al., 2005; Yuan et al., 2007). However, studies in rice and barley suggest that NPR1 function may not be fully conserved in monocots and dicots and that SA signaling and SAR in monocots might involve NPR1-independent pathways (Shimono et al., 2007; Dey et al., 2014). Transcription analysis in distal tissues revealed that bacteria-triggered SAR in barley was likely associated with jasmonic acid, ethylene and ABA, rather than SA. In contrast, SAR in maize is associated with SA accumulation in local and distal leaves (Balmer et al., 2013). Additionally, petiole exudates from pathogen infected *Arabidopsis* plants induced SAR in wheat (Chaturvedi et al., 2008). This suggests that SAR signaling in barley may not be similar to that in other monocots like maize and wheat.

The stability of NPR1 is dependent on Mediator (MED) 16 [allelic to Sensitive to Freezing (SFR) 6] (McKown et al., 1996; Warren et al., 1996), a subunit of the MED complex which functions as a bridge between transcription factors and the general RNA polymerase II transcriptional machinery (Zhang et al., 2012). A mutation in *MED16* compromises SAR and SA-induced defense responses but does not affect SA levels or nuclear localization of NPR1. Thus, MED16 likely functions downstream of SA in the SAR pathway. Interestingly, MED16 is also required for jasmonic acid/ethylene-responsive gene expression and resistance to necrotrophic pathogens (Zhang et al., 2012). Thus, MED16 might function by relaying signals from transcription factors that are specific to the SA and JA/ethylene pathways. A mutation in another MED subunit, MED 15 (isolated in a screen for non-recognition-of-the SA analog, BTH, *nrb4*), also attenuates SAR and SA responsiveness (Canet et al., 2012). However, MED15 is not required for NPR1 stability or localization and likely functions downstream of NPR1.

## SA versus Other SAR Inducers

Systemic acquired resistance is a complex phenomenon that involves the interplay of a diverse group of chemicals and associated proteins, besides SA. Most of these molecules can now be placed in one of two main branches that comprise the SAR pathway. One branch involves SA and its signaling component NPR1, and the other branch involves the free radicals NO and ROS, which function directly upstream of AzA, which in turn is upstream of G3P (Wang et al., 2014a; Wendehenne et al., 2014; El-Shetehy et al., 2015). Unlike G3P and AzA, exogenous application of Pip or DA induces SA accumulation in the absence of pathogen infection (Chaturvedi et al., 2012; Návarová et al., 2012). Therefore, Pip and DA likely function in the SA branch of SAR. The presence of two SAR branches is supported by the fact that SA cannot restore SAR in mutants defective in NO, ROS, or G3P biosynthesis, while NO/ROS cannot confer SAR on mutants defective in SA synthesis or signaling. Furthermore, pharmacological inhibitors of NO synthesis or NO scavengers attenuate SA-induced SAR in tobacco (Song and Goodman, 2001). Interestingly, unlike SA, both NO and ROS function in a concentration dependent manner because they can confer SAR only when present at an optimal concentration (Wang et al., 2014a). Free radicals are well known to operate similarly in animal systems where too little or too much can produce opposing physiological effects (Delledonne et al., 1998; Besson-Bard et al., 2008; Wink et al., 2011). Free radicals are thought to participate in SAR by mediating the oxidation of carbon (C) 18 unsaturated fatty acids (FAs) containing a double bond on C 9. This results in the formation of 9-oxo nonanoic acid (ONA), which is converted to the di-carboxylic acid AzA by the addition of a carboxylic group. AzA is unable to confer SAR on mutants unable to synthesize G3P, indicating it functions upstream of G3P. Exogenous AzA increases the expression of the G3P synthesizing *GLY1* and *GLI1* genes, which encode G3P dehydrogenase and glycerol kinase, respectively. G3P operates in a feedback loop with the LTPs DIR1 and AZI1 such that lack of DIR1 or AZI1 impairs pathogen-induced G3P accumulation while lack of G3P results in reduced *DIR1* and *AZI1* transcripts (Yu et al., 2013). DIR1 and AZI1 form homo- and hetromers suggesting that a complex comprising these proteins might function in SAR. Perhaps such a complex or the individual LTPs serve in transporting SAR essential signal(s) to the distal tissues. G3P appears to be the logical choice for such a transported signal since it is a precursor for lipid biogenesis. However, no direct interaction could be detected between G3P and DIR1 raising the possibility that G3P may be derivatized and this derivative may then be transported from infected to distal tissues. Radiolabel feeding experiments showed that G3P is indeed converted to an as yet unidentified derivative which can translocate from infected to distal tissues in a DIR1-dependent manner (Chanda et al., 2011).

Recent studies have shown that the C 18 FAs which serve as precusors for AzA are derived from the major plastidal lipids, monogalactosyldiacylglycerol (MGDG) and digalactosyldiacylglycerol (DGDG), which comprise ∼80% of the total lipids in plants (Zoeller et al., 2012; Gao et al., 2014). Thus, besides SA, NO, ROS and G3P, chloroplasts also serve as an important site for AzA biosynthesis. Notably, both galactose sugars in DGDG appear to be important for SAR since *dgd1* plants producing *α*-glucose-*β*-galactose diacylglycerol via transgenic expression of a bacterial glucosyltransferase, are not restored in SAR even though they are partially restored in chloroplast function. Thus it appears that the position of the hydroxyl group on C 4 of galactose may be important for SAR since glucose and galactose are sterioisomeric sugars which differ only in the position of their axial hydroxyl group at C 4.

## Cross Talk between SA and NO Pathways in SAR

Monogalactosyldiacylglycerol and DGDG galactolipids also serve additional functions in SAR. For instance, DGDG is required for SA and NO biosynthesis (Gao et al., 2014) and for AzA responsiveness. Interestingly, in spite of their impaired SA and NO synthesis, petiole exudates from pathogen-infected *dgd1* plants were able to confer SAR in wild-type plants. This suggests that *dgd1* plants can make signals that are capable of inducing SA- and NO-synthesis in plants with normal DGDG levels. These results show that SAR involves DGDG-dependent retrograde signaling between the chloroplast and nucleus and emphasizes the fact that the two branches of SAR are intricately linked (Gao et al., 2014).

In fact it is well known that there is cross talk between SA- and NO-mediated signaling. For example, NO mediated *S*-nitrosylation of NPR1 can result in the oligomerization and nuclear localization of NPR1 (Tada et al., 2008; Lindermayr et al., 2010). Moreover, SA has been suggested to regulate chloroplast structure since exogenous SA can cause swelling of grana thylakoids, coagulation of the stroma and increased chloroplast volume (Uzunova and Popova, 2000; Rivas-San Vicente and Plasencia, 2011). Regulation of SA and AzA levels by EDS1 is another case in point (Wittek et al., 2014). Together, these results suggest that the parallel operation of the interlinked SA- and NO-pathways might allow multiple points of regulation in fine tuning the optimal onset of SAR. This may be particularly relevant for signals like NO and ROS, which are functional within specific concentration ranges (Wang et al., 2014a).

## Conclusion and Perspectives

**FIGURE 2:**
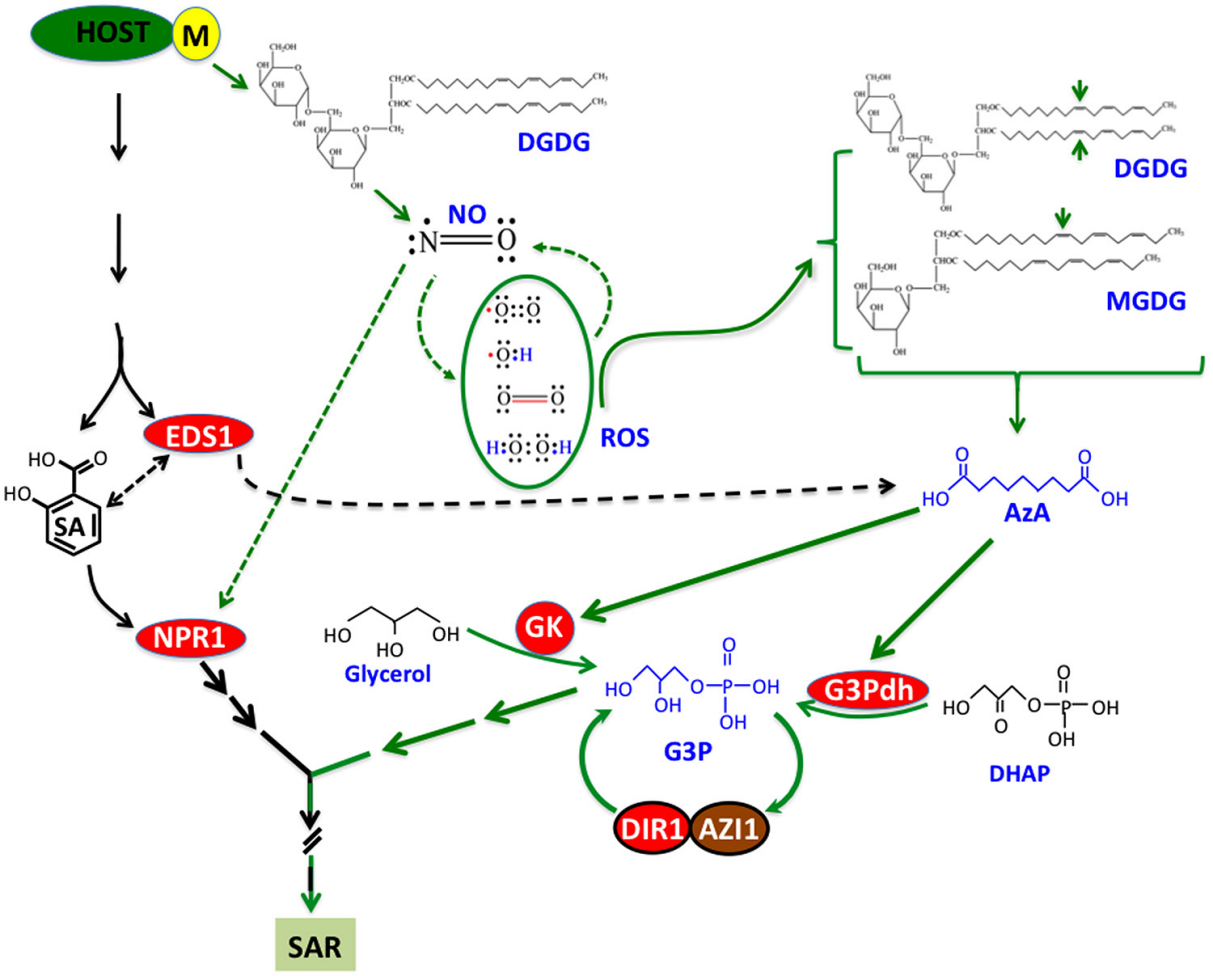
**A simplified model showing pathways, chemicals, and proteins involved in SAR**. Infection with avirulent (avr) pathogen induces accumulation of SA and nitric oxide (NO) in a digalactosyldiacylglycerol (DGDG)-dependent manner. NO operates in a feedback loop with reactive oxygen species (ROS), which catalyze oxidation of C18 unsaturated fatty acids (FA) present on monogalactosyldiacylglycerol (MGDG) and DGDG lipids. Oxidation of C18 FAs at C9 carbon (indicated by the arrows) generates azelaic acid (AzA), which triggers biosynthesis of glycerol-3-phosphate (G3P) via upregulation of genes encoding G3P biosynthetic enzymes (glycerol kinase, GK and G3P dehydrogenase, G3Pdh). G3P-mediated signaling is dependent on DIR1 and AZI1, which interact with each other and require G3P for the stability of their respective transcripts. Conversely, DIR1 and AZI1 are also required for G3P biosynthesis, suggesting that G3P and DIR1/AZI1 regulate SAR via a feedback loop. In the SA branch, EDS1 regulates both SA and AzA levels. NPR1 is a key downstream component in the SA branch which is nitrosylated by NO.

Recent work on SAR has identified a number of chemical and protein signals and placed them in a common pathway that comprises at least two parallel branches (**Figure 2**). However, these studies also indicate the involvement of additional unknown signal(s) that function upstream of the branchpoint separating SA-NPR1- and NO-ROS-AzA-G3P-derived pathways. In addition, several chemical signals, including G3P and AzA, undergo derivatization into unknown compounds and at least one of the G3P-derivative is SAR bioactive (unpublished data). Identification of these signals should provide useful insights into signaling events leading to the induction and establishment of SAR. Another area of SAR research that has not received much attention is the transport and perception of signals in the distal tissues. Although cuticle was implicated in the perception of SAR signals (Xia et al., 2009), later studies on cuticle mutants have suggested that perception might relate to the severity of cuticular damage or perhaps other unknown factors (Xia et al., 2012). These aspects of SAR should provide exciting avenues for studying how SAR overlaps with basic physiological processes and the distinct events that decide the onset of SAR versus normal growth and development.

## Conflict of Interest Statement

The authors declare that the research was conducted in the absence of any commercial or financial relationships that could be construed as a potential conflict of interest.
